# Adjunctive therapeutic plasma exchange as rescue therapy in septic shock due to streptococcal infection: a multicentric retrospective cohort study

**DOI:** 10.1186/s40635-026-00984-4

**Published:** 2026-10-28

**Authors:** Jannik Ruwisch, Alix Buhlmann, Lennart Wild, Pedro David Wendel-Garcia, Thorben Pape, Daniel A. Hofmaenner, Christian Putensen, Philippe Kruse, Greta-Henrike Holtgrave, Heiko Schenk, Christian Bode, Sascha David, Klaus Stahl, Benjamin Seeliger, the Bonn-Hannover-Zurich ARDS (BonHanZA) Study Group, Marius M. Hoeper, Konrad Peukert, Caroline Feuerborn, Finn Schirmer, Andrea Sauer, Mattia Mueller, Reto Schuepbach, Eva-Maria Kleinert, Rolf Erlebach, Rea Andermatt

**Affiliations:** 1 Department of Respiratory Medicine and Infectious Diseases, Hannover Medical School, Hannover, Germany; 2 Biomedical Research in End-Stage and Obstructive Lung Disease (BREATH), Hannover Medical School (MHH), German Center for Lung Research (DZL), Hannover, Germany; 3 PRACTIS Clinician Scientist Program, Dean’s Office for Academic Career Development, Hannover Medical School, Hannover, Germany; 4 Institute of Intensive Care Medicine, University and University Hospital Zurich, Zurich, Switzerland; 5 Department of Anaesthesiology and Intensive Care Medicine, University Hospital Bonn, Bonn, Germany; 6 Division of Cardiothoracic Anaesthesia and Intensive Care Medicine, Department of General Anaesthesia, Intensive Care and Pain Medicine, Medical University Vienna, Vienna, Austria; 7 Department of Nephrology and Hypertension, Hannover Medical School, Hannover, Germany; 8 Department of Gastroenterology, Hepatology, Infectious Diseases and Endocrinology, Hannover Medical School, Hannover, Germany

**Keywords:** Septic shock, Sepsis, Streptococcus, Therapeutic plasma exchange, Mortality

## Abstract

**Background:**

Septic shock due to streptococcal infection (SRSS) carries a high mortality and is in part driven by toxin-mediated inflammation. Therapeutic plasma exchange (TPE) may modulate the septic host responses by removing injurious mediators while replenishing protective plasma factors, but data specific to SRSS is lacking. We performed a retrospective, multicenter, cohort study of adults with SRSS between 2012–2024.

**Methods:**

Patients from three tertiary care ECMO centers in Germany and Switzerland were included requiring (i) streptococcal spp. detected in blood or primary focus and (ii) septic shock according to sepsis-3 definition. Adjunctive TPE with fresh frozen plasma at the intensivists` discretion was compared with standard of care (SOC). The primary outcome was 90-day-hospital mortality. Key exploratory outcomes included 24-h kinetics of vasoactive-inotropic score(VIS) and lactate, as well as SRSS-related complications. Results were validated in a 1:1 propensity score–matched (PSM) cohort.

**Results:**

Of 149 patients, 31 (20.8%) received TPE and 118 (79.2%) SOC. TPE patients were more severely ill at baseline: SOFA 14 (IQR 13.5–15) vs 11 (IQR 8–14) (p<0.001); lactate 6.4 vs 2.8 mmol/L (p<0.001); Vasoactive-Inotropic-Score (VIS) 70.7 vs 23.3 (p<0.001); more frequent invasive ventilation (87% vs 65%), VV-ECMO (36% vs 19%), and renal replacement therapy (96.8% vs 52.5%). Overall, 90-day hospital mortality was 27.5% (TPE 29.0%, SOC 27.1%). Following SOFA-score adjustment, TPE was associated with lower mortality risk (aHR 0.67 [95% CI 0.5–0.9]; p=0.009). This was validated in the PSM cohort, with an aHR for TPE of 0.44 (95% CI 0.3–0.8); p=0.002. Mixed-effects modeling demonstrated a significant VIS reduction only with TPE (fixed effect 24-h change, p=0.027) and improved lactate decrease versus SOC.

**Conclusion:**

In SRSS, adjunctive TPE was associated with rapid hemodynamic stabilization and improved lactate kinetics. Exploratory adjusted survival analyses support prospective evaluation of TPE in SRSS.

**Supplementary Information:**

The online version contains supplementary material available at https://doi.org/10.1186/s40635-026-00984-4.

## Background

Septic shock is a life-threatening organ dysfunction due to dysregulated host responses to infection harboring significant mortality [1]. The host response is characterized by immune, endothelial and coagulation alterations. The immune response patterns in septic shock are highly variable and recently several clinical phenotypes with distinct treatment response to adjunct interventions have been identified [2, 3]. In septic shock, exposure to bacterial toxins may result in both complement and cytokine activation resulting in a hyperinflammatory phenotype associated with highest rates of mortality [2, 4].

Streptococci are a common cause of septic shock accounting for to up to 13.5% of identified pathogens in hospital admissions with sepsis [5] and up to 20% of isolates in intensive care unit (ICU) blood stream infections [6]. Streptococcal strains produce diverse virulence factors; most strikingly, S. pyogenes (Group A Streptococci)[7] and S. pneumoniae [8]. The role of extracorporeal removal specifically of streptococcal toxins is unclear and limited to case reports using therapeutic plasma exchange (TPE) [9–11] and broad-spectrum hemoadsorption [12–14].

In the past, TPE has re-emerged as a promising adjunctive treatment in septic shock with recent aggregation of evidence demonstrating its ameliorating effect on organ dysfunction including reduction of vasopressor dose [15, 16]. TPE may be particularly helpful in SRSS because of its potential to firstly reduce circulatory acute-phase proteins [17] and chemo-attractants [18] and to secondly rebalance deranged coagulation states including disseminated intravascular coagulation [19, 20] and thirdly, restore endothelial barrier function [15, 21, 22]. The experience derived from prior studies might not be specific to streptococcal infection but systematic analyses are lacking. With incidence of group-A streptococcal infections on the rise [23–29], data about possible adjunctive therapies in severe disease are warranted.

This study aimed to retrospectively aggregate the pragmatic clinical experience with TPE in SRSS in three university ICUs.

## Methods

### Patient selection and study design

**Fig. 1:**
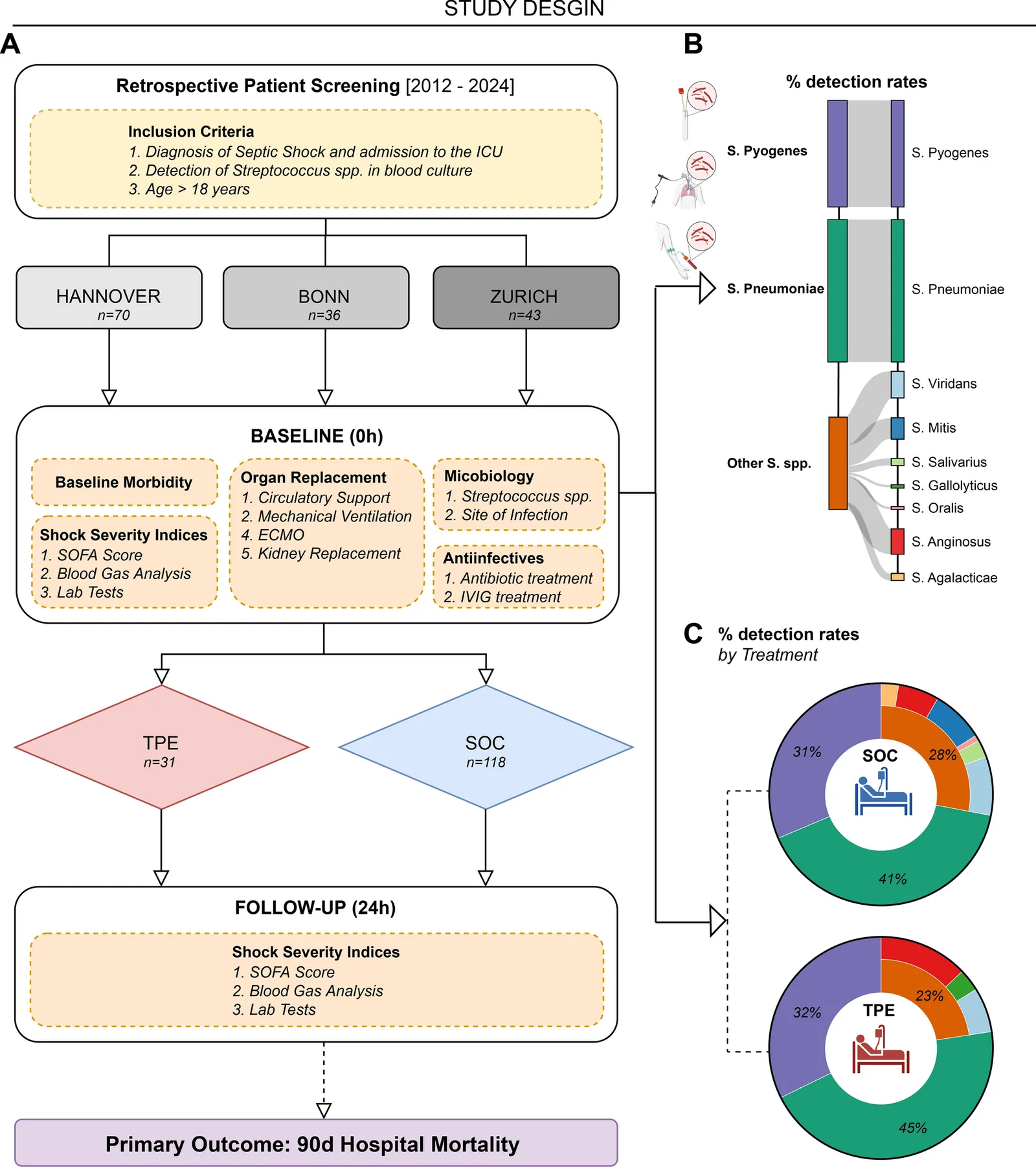
Study workflow and identified streptococcus isolates. The study scheme is provided in panel **(A)**. Relative frequencies of detected streptococcus species are provided as distorted bar plot **(B)**. Ring plots denote relative streptococcus isolate frequencies split by treatment strata **(C). **SOFA: sequential organ failure assessment; ECMO: extracorporeal membrane oxygenation; spp.: species; IVIG: intravenous immunoglobulins; TPE: therapeutic plasma exchange; SOC: standard of care; ICU: intensive care unit; IVIG: intravenous immunoglobulins and S: streptococcus

In this retrospective multicenter, binational observational cohort study, we investigated patients with streptococcus-related septic shock that had been admitted to the intensive care unit of the three study sites in Hannover, Bonn and Zurich in Germany and Switzerland, respectively between 2012–2024 (Fig. 1A). The study was reviewed and approved by the local institutional review boards of all participating institutions: 11221_BO_K_2024 (Hannover); BASEC 2024–00993 (Zurich); 437/23-EP (Bonn).

Patients were eligible for the study when they had the clinical picture of septic shock in relation to a streptococcal infection defined as i) detection of streptococcus spp. (*not enterococcal* [30]) in either blood culture or site of primary infection and ii) septic shock according to the SEPSIS 3 criteria [31]. Within this study, they were labelled as “Streptococcal related septic shock” (SRSS). Isolation of toxigenic strains related to streptococcus toxic shock and/or streptococcus scalded skin syndrome was not considered a prerequisite for study inclusion, as superantigen typing was not routinely assessed. Definition of streptococcus toxic shock syndrome (STSS) was adapted from the working group definition [32]. Patients < 18 years of age were excluded from the study. At the Zurich site, TPE was not part of local institutional practice during the study period. Patients from this center were thus retained for the SOC group to mitigate selection bias. An a priori power size calculation was not performed given the retrospective nature of this study.

### Investigational treatment: TPE vs SOC

Precise criteria for TPE use were not protocolized, and TPE was initiated at the intensivists’ discretion. Generally, it was considered for patients with increasing norepinephrine doses > 0.4 µg/kg/min or ≤ 0.4 µg/kg/min but increasing lactate levels as rescue therapy (in the following labelled as “TPE-group”). TPE was performed via an 11-French two-lumen hemodialysis catheter in the internal jugular or femoral vein, exchanging a fixed dose of 12 units of fresh frozen plasma per session over approximately 2 h at a blood flow of about 60 mL/min under regional citrate anticoagulation. A centrifugal system was used in Hannover (Spectra Optia, Terumo) and a membrane-based system in Bonn (multiFiltrate, Fresenius), without additional pathogen inactivation [16]. TPE was usually performed once but was repeated as per individual clinical judgement. Patients who did not receive TPE regardless of severity of SRSS were labelled “SOC”.

### Primary and key exploratory outcomes

90-day hospital mortality was predefined as the primary outcome. Therefore, patients were followed until death and censored at hospital discharge or transfer to rehabilitation. Key exploratory outcomes included trajectories of serum lactate and VIS. Adverse events of special interest included limb ischemia, limb amputation, need for infection-related intervention, occurrence of pleural empyema or septic peripheral embolism in any organ. Patients were stratified by having received TPE as rescue therapy for persistent septic shock or SOC compliant with current national [33] and international recommendations [34].

### Clinical definitions and data analysis

Throughout the study we defined two distinct time points, namely the time of ICU admission (baseline, 0 h) and a first follow-up time point after 24 h starting from admission (follow-up, 24 h, Fig. 1A). Shock severity indices by means of sequential organ failure assessment (SOFA) score, serum lactate and vasopressors levels were evaluated at both time points. Vasopressor levels were calculated as weighted VIS as previously described [35], integrating administration rates of epinephrin, norepinephrine, dobutamine and vasopressin. Patients with decreasing VIS levels between baseline and 24 h of follow-up (defined as any ΔVIS<0) were subsequently labeled as “responders” to either SOC or TPE treatment.

Descriptive statistics of categorical and discrete variables at baseline were analyzed by Chi-squared test, while continuous clinical variables were tested by Wilcoxon’s Rank Sum test for non-parametric variables and paired t-test for paired parametric variables. Normality was assessed using the Shapiro–Wilk test. VIS score was logarithmized in the analysis to achieve normal distribution.

90-day hospital mortality was evaluated by Kaplan–Meier testing and a bivariate site-clustered Cox regression model adjusting for SOFA-Score at baseline. Patients who were transferred to a rehabilitation facility were censored in the survival analyses at the time of transfer.

For the primary analysis, results from the survival analysis were validated on a 1:1 PSM cohort (optimal matching) accounting for age, sex, VIS [35], SOFA score and lactate levels at baseline, streptococcus-subspecies group (*S. pneumoniae, S. pyogenes,* other, Fig. 1B,C), and prior immunosuppression. For the two centers which used TPE, only patients before 2021 were used for matching because after the publication of the EXCHANGE−1 results [16], TPE transitioned from experimental treatment to established therapy in these centers. Patients thereafter were thus excluded to prevent confounding by indication. The standardized mean difference was calculated for each variable to evaluate resultant balance statistics. Covariate balance was visualized as love plots.

Three sensitivity analyses were performed. In the first, the species group was replaced by the primary source of infection and invasive ventilation, renal replacement therapy and ECMO at baseline were added to the propensity score model. In the second, procalcitonin, hydrocortisone and adjunctive clindamycin therapy were added. In the third, the Zurich center, at which TPE was not available, was excluded from matching. The primary propensity score model achieved the best covariate balance and was therefore retained for the primary analyses.

Effects of TPE on lactate and VIS trajectories and occurrences of secondary SRSS related complications were analyzed using linear mixed-effects models accounting for random site-related inter-subject variation as well as treatment group as fixed effect. All analyses were conducted using the R environment for statistical computing version 4.1.2 (R Foundation for Statistical Computing, Vienna, Austria).

## Results

### Patient population

A total of 149 patients suffering from SRSS were identified at three ICUs between 2012 and 2024 in Germany (Hannover, n=70; Bonn n=36) and Switzerland (Zurich, n=43). Herein, 31 patients (20.8%) had received at least one session of adjunctive TPE with a median time from ICU admission to TPE initiation of 8.1 h, while 118 (79.2%) patients had been subjected to SOC without TPE (Fig. 1A**, **Table 1). Most patients received a single TPE session (25/31, 81%) and in 6 patients (19%), TPE was repeated with a mean of 2.5 sessions.

**Table 1:** Baseline demographics by treatment group

Variable (median [IQR] or N [%])	SOC (N=118)	TPE (N=31)	Total (N=149)	*p*-Value
**Age [y]**	54.4 (41.2, 67.3)	43.9 (40.9, 58.5)	53.6 (40.9, 66.6)	**0.027**^**#**^
**Sex (female)**	49 (41.5%)	13 (41.9%)	62 (41.6%)	0.967^‡^
**BMI**	24.7 (22.9, 31.1)	25.9 (23.3, 28.9)	25.2 (22.9, 30.9)	0.894^#^
**Comorbidities**				
*Congestive heart failure*	40 (33.9%)	3 (9.7%)	43 (28.9%)	**0.008**^**‡**^
*Cardiovascular disease*	45 (38.1%)	5 (16.1%)	50 (33.6%)	**0.021**^**‡**^
*Diabetes mellitus*	26 (22.0%)	6 (19.4%)	32 (21.5%)	0.747^‡^
*Chronic kidney disease*	25 (21.2%)	3 (9.7%)	28 (18.8%)	0.144^‡^
**Primary Site of Infection**				0.402^‡^
*Lung*	48 (40.7%)	19 (61.3%)	67 (45.0%)	
*Soft tissue*	35 (29.7%)	5 (16.1%)	40 (26.8%)	
*Abdominal*	12 (10.2%)	4 (12.9%)	16 (10.7%)	
Other*	23 (19.4%)	3 (9.7%)	26 (17.5%)	
**SOFA Score (admission)**	11 (8, 14)	14 (13.5, 15)	12 (9, 14)	**<0.001**^**#**^
**CRP**_**0h**_** [mg/l]**	224 (110, 327)	180 (124, 290)	218 (120, 319)	0.956^#^
**Procalcitonin**_**0h**_** [µg/l]**	18.3 (4.0, 75.2)	73.4 (13.5, 203.0)	24.900 (5.8, 95.2)	**0.006**^**#**^
**Lactate [mmol/l]**	2.8 (1.6, 5.2)	6.4 (4.5, 8.2)	3.7 (2, 6.3)	**<0.001**^**#**^
**Organ support**				
*VIS*_*0h*_	23.3 (10.8, 44.9)	70.7 (53.1, 123.8)	31 (12.4, 64.8)	**<0.001**^**#**^
*Renal replacement therapy*	62 (52.5%)	30 (96.8%)	92 (61.7%)	**<0.001**^**‡**^
*VV-ECMO*	22 (18.6%)	11 (35.5%)	33 (22.1%)	**0.044**^**‡**^
*Mechanical ventilation*	77 (65.3%)	27 (87.1%)	104 (69.8%)	**0.018**^**‡**^
**Treatment Modalities**				
*Time to TPE [h]*	NA	8.1 (4.5, 21.2)	NA	
*Clindamycin*	37 (31.4%)	14 (45.2%)	51 (34.2%)	0.149^‡^
*IVIG*	20 (16.9%)	5 (16.1%)	25 (16.8%)	0.913^‡^
*Steroids*^*+*^	63 (53.4%)	29 (93.5%)	92 (61.7%)	**<0.001**^**‡**^
**Site**				**<0.001**
*Bonn*	30 (25.4%)	6 (19.4%)	36 (24.2%)	
*Hannover*	45 (38.1%)	25 (80.6%)	70 (47.0%)	
*Zurich*	43 (36.4%)	0 (0.0%)	43 (28.9%)	
**Hospital Death (90d)**	32 (27.1%)	9 (29.0%)	41 (27.5%)	0.832^‡^
Length ICU* [d]	10 (5, 24)	29 (10, 47)	11 (6, 29)	**0.003**^**#**^
**Vasopressor-free days**	3 (0, 9)	3 (0, 17)	3 (0, 11)	0.54
**Ventilator-free days**	2 (0, 6)	0 (0, 5)	2 (0, 6)	**0.058**
**Status Amputation**	5 (4.2%)	4 (12.9%)	9 (6.0%)	0.071^‡^
**Status Intervention**	39 (33.1%)	8 (25.8%)	47 (31.5%)	0.44^‡^
**Status limb ischemia**	13 (11.0%)	9 (29.0%)	22 (14.8%)	**0.012**^**‡**^
**Status pleural empyema**	7 (5.9%)	3 (9.7%)	10 (6.7%)	0.458^‡^
**Status septic embolism**	16 (13.6%)	4 (12.9%)	20 (13.4%)	0.924^‡^

BMI: body mass index; CRP: C-reactive protein; Bonn: University Hospital Bonn; Hannover: Hannover Medical School; Zurich: University hospital Zurich; VV-ECMO: veno-venous extracorporeal membrane oxygenation; IVIG: intravenous immunoglobulins. #=Wilcoxon-Rank Sum test; ‡=Chi-Squared test. *Other sites of infection include heart, ear-nose-throat, lymph node, central nervous system and kidneys ^+^200 mg hydrocortisone for septic shock

Bold *p*-values indicate statistical significance (*p* < 0.05)

The median age in the overall cohort was 54 years (IQR 41–67), while TPE patients were significantly younger (44 years [IQR 41–59], Table 1) than patients who received SOC (54 years [IQR 41–67]). Slight male predominance was noted with 58.4% in the pooled cohort. Regarding the assessed comorbidities, only a significant underrepresentation of chronic heart failure (9.7% vs. 33.9%, p=0.008) and cardiovascular disease in the TPE group (16.1% vs. 38.1%, p=0.021) was noted. Of note, baseline C-reactive protein (CRP) levels did not differ among treatment groups (p=0.956), while baseline procalcitonin levels (PCT) were significantly higher in patients treated with TPE (73.4 µg/l, [IQR 13.5–203] vs. 18.3 µg/l [IQR 4.0–75.2], p=0.006).

### TPE was initiated in more severe SRSS

Median SOFA scores were significantly higher at admission with a median of 14 points [IQR 13.5–15] in the TPE group vs. a median of 11 points [IQR 8–14] in the SOC group (p<0.001) **(**Table 1**)**. A similar trend was observed for baseline arterial lactate levels, suggesting a more compromised organ perfusion or microcirculation in the TPE group (6.4 mmol/L [IQR 4.5–8.2] vs. 2.8 mmol/L [IQR 1.6–5.2], p<0.001). Moreover, VIS was significantly higher in the TPE group (70.7 [IQR 53.1–123.8] vs. 23.3 [IQR 10.8–44.9], p<0.001), further indicating a more pronounced macrovascular circulatory dysfunction. Overall, artificial organ support was more extensively required at baseline in the TPE group including a higher need for mechanical ventilation (TPE: 87% vs. SOC: 65%, p=0.018), veno-venous (VV)-ECMO, TPE: 36% vs. SOC 19%, p=0.044), and renal replacement therapy (TPE: 96.8% vs. SOC: 52.5%, p<0.001) in the TPE group. In regard to hemostatic parameters, platelet counts declined within the first 24 h in both groups, more markedly after TPE (104 to 62 ×10^9^/l, p<0.001) than under SOC (129 to 106 ×10^9^/l, p=0.002). Baseline D-dimers did not differ between groups (12.6 vs. 7.3 mg/l, p=0.088, Fig. S1).

### Identification of streptococcal isolates, antimicrobial and adjunctive therapy

The most frequently detected streptococcus species (spp.) in blood culture or site of primary infection were *S. pneumoniae* (45% vs. 41%, Fig. 1B-C) followed by *S. pyogenes* (31% vs. 32%, Fig. 1B-C). Of the 47 patients with *S. pyogenes* infection, 37 fulfilled the clinical criteria of STSS (32.3% vs 22.9%, p=0.35) [32]. Other identified species (23% vs. 28%) comprised *S. viridans*, *S. mitis*, *S. salivarius*, *S. gallolyticus*, *S. oralis*, *S. anginosus* and *S. agalactiae*. Primary site of infection did not differ among groups (p=0.402) with lung (n=67, 45%) soft tissue (n=40, 27%) and abdominal (n=16, 11%) sites being most common (Table 1).

All patients received adequate anti-streptococcal broad-spectrum antibiotics with 88% receiving either piperacillin-tazobactam (60%), meropenem (51%) or ampicillin-sulbactam (8%). 34% of all patients subsequently received either targeted therapy with penicillin G or additional penicillin G during ICU treatment. With the intention of suppressing toxin production, 34% received additional clindamycin (Table 1).

Intravenous immunoglobulins were administered at similar rates (16.1% vs. 16.9%) and steroids (hydrocortisone 200 mg over 24 h for septic shock) had been more frequently given in the TPE group (93.5% vs. 53.4%, p<0.001).

### TPE was associated with lower adjusted 90-day hospital mortality in patients with SRSS

**Fig. 2.**
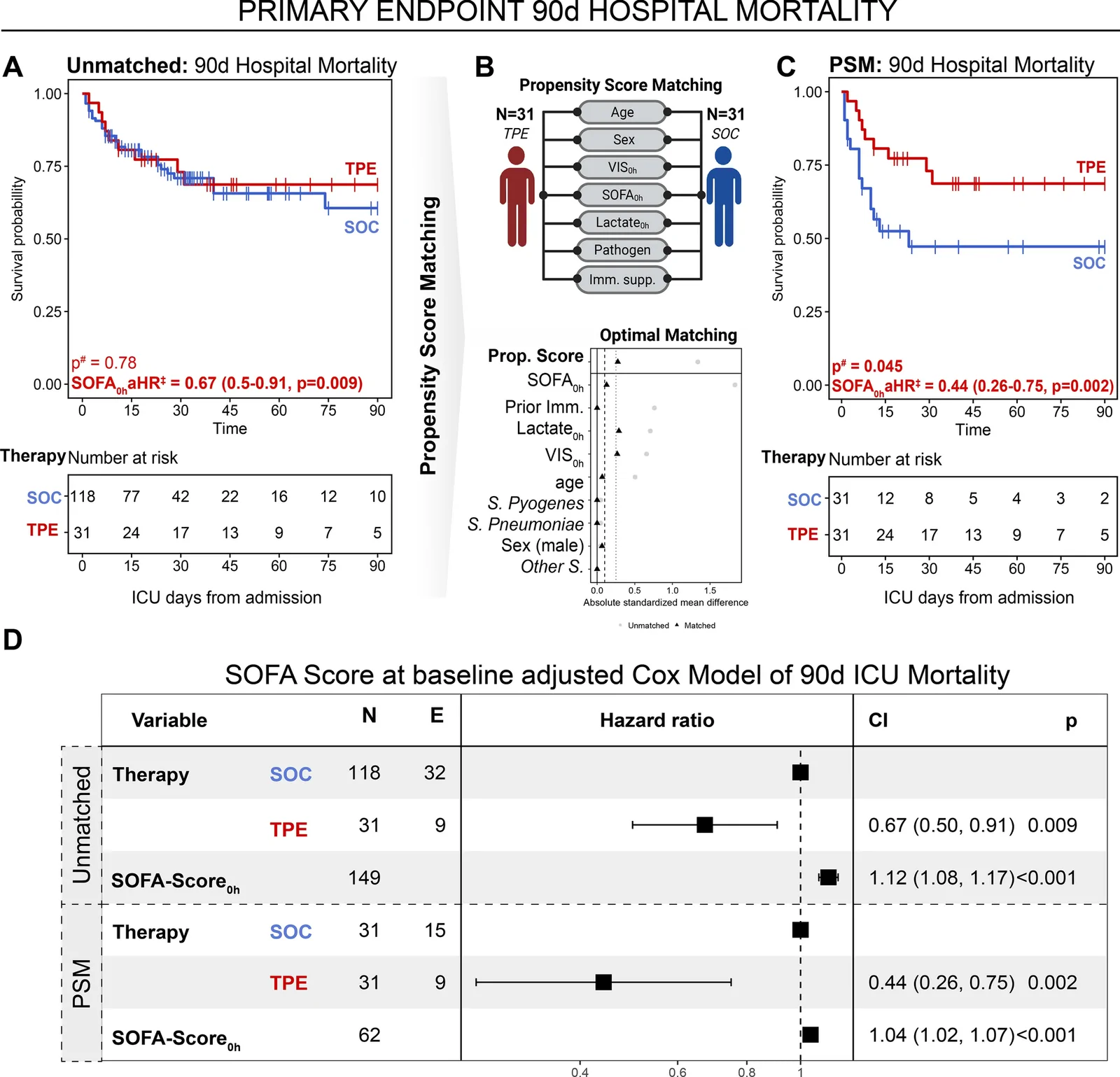
90-day hospital mortality analysis. The primary outcome of 90d-hospital mortality was analyzed for the pooled cohort enrolled at all three study sites and was stratified by TPE vs. SOC. Unmatched survival analysis showed no survival benefit for TPE by log-rank test **(A)**. TPE-treated patients had improved adjusted survival in the baseline SOFA score (SOFA_0h_) adjusted cox regression analysis. Validation was performed via optimal propensity score matching (PSM) for age, sex, VIS_0h_, SOFA_0h_, lactate_0h_, pathogen-group and prior immunosuppression. Absolute standardized mean differences before and after PSM are shown in **(B)**. The dashed line indicates the conventional balance threshold of 0.1, the dotted line the more lenient criterion of 0.25. PSM matched survival analysis showed improved survival for TPE in the Kaplan–Meier and SOFA_0h_ adjusted cox regression analysis **(C)**. Effect size comparison of the PSM matched and unmatched cox regression models demonstrated greater reduction in mortality in the PSM-matched analysis **(D). **TPE: therapeutic plasma exchange; SOC: standard of care; SOFA_0h_: sequential organ failure assessment at baseline; aHR: adjusted hazard ratio; Lactate_0h_: lactate at baseline; VIS_0h_: vasoactive inotropy score at baseline; Imm. Supp/Imm.: immunosuppression at baseline; Prop. Score: Propensity Score; PSM: propensity score matched; N: number of patients; E: number of events and CI: confidence interval. #=log-rank test; ‡=SOFA_0h_ adjusted cox-regression model

90-day hospital survival was assessed stratified by treatment groups in the pooled cohort of all study sites. Overall, 90d-hospital mortality was 27.5% (29% in TPE; 27.1% in SOC). Kaplan–Meier analysis showed no significant mortality differences between TPE and SOC (p=0.78), Fig. 2A). However, after adjustment for baseline severity through baseline SOFA (SOFA_0h_) score TPE was highly associated with reduced mortality with an adjusted hazard ratio (aHR) of 0.67 (95% CI [0.5–0.9], p=0.009, Fig. 2A).

For further validation, PSM was performed matching TPE patients 1:1 to patients from the SOC group (Fig. 2B), resulting in n=31 TPE patients matched with n=31 SOC patients. Due to the excessive disease severity in the TPE group, despite matching, overall organ support by means of renal replacement therapy and VIS were still significantly higher in the TPE group, although differences were clearly reduced (Fig. 2B, Table 2). Log-rank analysis revealed a statistically significant reduction in 90-day hospital mortality among patients in the TPE group (p=0.045, Fig. 2C). Computation of 90-day hospital mortality employing the same SOFA_0h_ adjusted model demonstrated an augmented protective effect in the TPE group (aHR=0.44, 95% CI [0.3–0.8], p=0.002, Fig. 2D).

**Table 2:** Baseline demographics of propensity score matched patient populations

Variable (median [IQR] or N [%])	SOC (N=31)	TPE (N=31)	Total (N=62)	*p*-Value
**Age [y]**	45 (38, 57)	44 (41, 59)	44 (38, 59)	0.983^#^
**Sex (female)**	14 (45.2%)	13 (41.9%)	27 (43.5%)	0.798^‡^
**BMI**	25.5 (23.7, 29.2)	25.9 (23.3, 28.9)	25.7 (23.4, 29.3)	0.966^#^
**Comorbidities**				
*Congestive heart failure*	6 (19.4%)	3 (9.7%)	9 (14.5%)	0.279^‡^
*Cardiovascular disease*	5 (16.1%)	5 (16.1%)	10 (16.1%)	1^‡^
*Diabetes mellitus*	4 (12.9%)	6 (19.4%)	10 (16.1%)	0.49^‡^
*Chronic kidney disease*	3 (9.7%)	3 (9.7%)	6 (9.7%)	1^‡^
**Primary Site of Infection**				0.656^‡^
*Lung*	15 (48.4%)	19 (61.3%)	34 (54.8%)	
*Soft tissue*	9 (29.0%)	5 (16.1%)	14 (22.6%)	
*Abdominal*	4 (12.9%)	4 (12.9%)	8 (12.9%)	
Other*	3 (9.7%)	3 (9.7%)	6 (9.7%)	
**SOFA Score**	15 (13, 17)	14 (14, 15)	14 (13, 16)	0.409^#^
**CRP**_**0h**_** [mg/l]**	199.9 (103.3, 261.5)	180 (124, 290)	196.9(120, 278)	0.619^#^
**Procalcitonin**_**0 h**_** [µg/l]**	18.8 (6.2, 55.9)	73.4 (13.5, 203)	40.7(8.3, 139.8)	**0.04**^**#**^
**Lactate [mmol/l]**	4.6 (2.4, 8.5)	6.4 (4.5, 8.2)	5.9 (3.6, 8.5)	0.081^#^
**Organ support**				
*VIS*_*0h*_	40 (16.1, 71.4)	70.7 (53.1, 123.8)	61.3 (31.6, 93.5)	**0.012**^**#**^
*Renal replacement therapy*	23 (74.2%)	30 (96.8%)	53 (85.5%)	**0.012**^**‡**^
*VV-ECMO*	9 (29.0%)	11 (35.5%)	20 (32.3%)	0.587^‡^
*Mechanical Ventilation*	25 (80.6%)	27 (87.1%)	52 (83.9%)	0.490^‡^
**Treatment Modalities**				
*Clindamycin*	11 (35.5%)	14 (45.2%)	25 (40.3%)	0.437
*IVIG*	4 (12.9%)	5 (16.1%)	9 (14.5%)	0.718^‡^
*Steroids*^*#*^	19 (61.3%)	29 (93.5%)	48 (77.4%)	**0.002**^**‡**^
**Site**				**<0.001**^**‡**^
*Bonn*^*+*^	6 (19.4%)	6 (19.4%)	12 (19.4%)	
*Hannover*^*+*^	13 (41.9%)	25 (80.6%)	38 (61.3%)	
*Zurich*	12 (38.7%)	0 (0.0%)	12 (19.4%)	
**Hospital Death (90d)**	15 (48.4%)	9 (29.0%)	24 (38.7%)	0.118^‡^
Length ICU* [d]	7 (3, 20)	29 (10, 47)	12 (5, 38)	**0.005**^**#**^
**Vasopressor-free days**	2 (0, 6)	3 (0, 17)	2 (0, 11)	0.301
**Ventilator-free days**	1 (0, 4)	0 (0, 5)	0 (0, 5)	0.355
**Status Amputation**	2 (6.5%)	4 (12.9%)	6 (9.7%)	0.39^‡^
**Status Intervention**	9 (29.0%)	8 (25.8%)	17 (27.4%)	0.776^‡^
**Status limb ischemia**	3 (9.7%)	9 (29.0%)	12 (19.4%)	0.054^‡^
**Status pleural empyema**	4 (12.9%)	3 (9.7%)	7 (11.3%)	0.688^‡^
**Status septic embolism**	2 (6.5%)	4 (12.9%)	6 (9.7%)	0.390^‡^

BMI: body mass index; CRP: C-reactive protein; Bonn: University Hospital Bonn; Hannover: Hannover Medical School; Zurich: University Hospital Zurich; VV-ECMO: veno-venous extracorporeal membrane oxygenation; IVIG: intravenous immunoglobulins. #=Wilcoxon-Rank Sum test; ‡=Chi-Squared test. *Other sites of infection include heart, ear-nose-throat, lymph node, central nervous system and kidneys ^+^Only patients admitted to the ICU prior to 2021 were considered for propensity score matching ^#^200 mg hydrocortisone for septic shock

Bold *p*-values indicate statistical significance (*p* < 0.05)

### Sensitivity analyses for alternative PSM strategies

The effect estimate was attenuated but still significant when the primary propensity score model additionally accounted for hydrocortisone and clindamycin (aHR 0.62, 95% CI [0.4–0.9], p=0.014, Fig. S2), while a second PSM sensitivity analysis including organ replacement therapy and site of infection as variates yielded a similar effect estimate (aHR = 0.33 95% CI [0.2–0.6], p<0.001). In a third sensitivity analysis, excluding the Zurich center from PSM, the reduction in 90-day hospital mortality with TPE was preserved in both the log-rank test (p=0.04) and the SOFA_0h_ adjusted Cox regression (aHR of 0.37 95% CI [0.3–0.4], p<0.001, Fig. S3).

### Secondary SRSS-related outcomes were unaffected by TPE

Length of ICU stay was substantially longer in the TPE group (29d [IQR 10–47] vs. 10 d [IQR 5–24], p=0.003, Table 1). Regarding complications of streptococcal infection (acral necrosis, amputation, need for intervention, pleural empyema and septic embolism), acral necrosis was found to be more prevalent in the TPE group (TPE: 29% vs. SOC: 11%,. p=0.012), while comparative analysis of need for limb amputation only exhibited a numerical difference (12.9% vs. 4.2%, p=0.071). Of note, these differences were no longer present when comparing the PSM-cohorts **(**Table 2**)** and none of these adverse events were deemed to be a consequence of TPE. Meanwhile, TPE exhibited no protective effects on any of the assessed exploratory outcomes (Table 2).

### Treatment responsiveness by vasopressor administration predicts survival

Further, early predictors for improved outcomes within the TPE-treated sub-cohort were assessed. We tested whether an early reduction in VIS (after 24 h), referred to as “TPE-responders” may also reflect overall survival (Fig. S4A). VIS-response was seen in 74% within the TPE group vs. 64% in the SOC group. Non-responders had increased 90-day hospital mortality compared to responders (Hazard Ratio [HR] 2.68, 95%-Confidence Interval [CI] [1.6–4.4], p<0.001, Fig. S4B-D). Vice versa, TPE treated SRSS survivors showed significantly higher absolute early VIS reduction (*p*=0.005), while 90 d non-survivors, exhibited no such decline (p=0.479) (Fig. S4C). A similar effect on 90 d hospital mortality was observed in non-responders to SOC (HR=5.27; 95%-CI 1.3–21.29, p=0.02, Fig. S4E-G).

### TPE was associated with improved VIS and lactate reduction

**Fig. 3:**
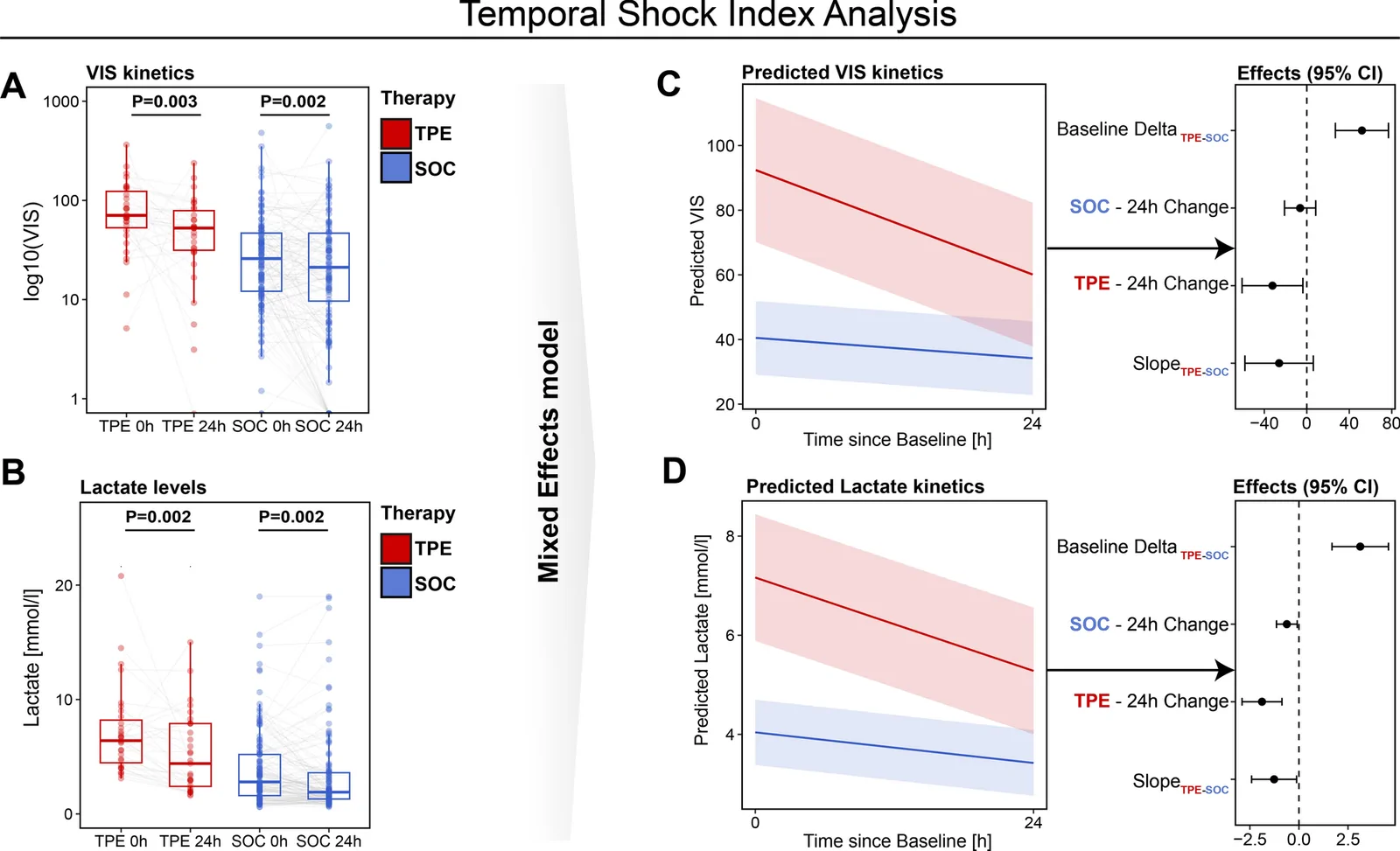
Temporal kinetics of shock indices. Temporal VIS kinetics throughout the initial 24 h upon ICU admission showed a significant decrease in both treatment groups (**A**). Temporal lactate levels mirrored kinetics of VIS with significant decreases in both groups **(B)**. Mixed effects modelling of VIS score kinetics showed a sharper predicted decline in the TPE groups **(C)**. Fixed effect (slope) was only significant for the TPE groups (TPE – 24 h change), but not the SOC group, while baseline intercept was significantly elevated in the TPE group. Lactate kinetics as predicted by mixed effects modelling showed a significant decline in both treatment groups (SOC – 24 Change, TPE−24 – Change), while this fixed effect was significantly higher in the TPE group (Slope_TPE-SOC_) **(D)**. VIS levels were logarithmized. P-values of paired differential analysis were calculated by Wilcoxon signed-rank test. Forest plots denote fixed effects and related intercepts of mixed-effect model VIS: vasoactive inotropy score; Predicted VIS: VIS levels predicted by mixed-effect model; predicted lactate: and lactate levels predicted by mixed-effect model

Temporal analysis between baseline and 24 h showed significant declines in VIS and lactate levels (Fig. 3A, B) for both groups. Within the PSM cohort, a significant reduction in VIS and lactate levels was only observed in patients receiving adjunctive TPE (Fig. S5). In line, mixed effects modeling of VIS and lactate not only revealed a significantly higher baseline value (intercept) in the TPE group for both variables (Fig. 3C, D), but also illustrates a stronger predicted reduction of VIS after 24 h in the TPE group (fixed effect: − 32.3, 95%-CI [− 60.9,−3.7], TPE – 24 h change, *p*=0.027, Fig. 3C). This association was not statistically significant in the SOC group (SOC – 24 h change: − 6.3, p=0.399, Fig. 3C). For lactate kinetics **(**Fig. 3D**)**, the baseline lactate levels (intercept) were higher and the absolute lactate reduction after 24 h was increased in the TPE group with a significantly greater reduction over time (Slope_TPE-SOC_: − 1.3 mmol/L, 95%-CI [− 2.4, − 0.1], p=0.031, Fig. 3D).

## Discussion

Previously confined to case series, this is the first multicentric analysis to systematically describe the effect of adjunctive TPE in SRSS.

TPE treated patients showed significantly aggravated organ-dysfunction at baseline indicated by overall higher SOFA-score, vasopressor administration, renal replacement therapy, mechanical ventilation and ECMO support. Still, mortality was numerically similar between the two groups and SOFA-adjusted Cox modelling favored adjunctive TPE. This finding was even more striking after PSM. Baseline lactate remained numerically higher in the TPE cohort after matching. Residual imbalance therefore persisted in the direction of greater severity in the TPE group.

Recently, the clinical and physiological effects of TPE, employed as an experimental adjunctive treatment in refractory shock patients, have been studied in depth by our group and others. A pilot trial and the randomized Exchange−1 study demonstrated a significant reduction of vasopressor administration with TPE [15, 16] and recent meta- and network analyses have even suggested potential survival benefits with TPE in septic shock [36, 37]. Aligning with these previous observations, herein we also observed a marked improvement of vasopressor administration and lactate kinetics with TPE over SOC. Recent evidence confirmed a close association of norepinephrine dose with mortality in critically ill patients, reinforcing the role of norepinephrine doses as an indicator of cardiovascular failure severity [38]. Of note, early vasopressor dose de-escalation within the first 24 h was closely associated with improved 90-day survival in this study both in the TPE as well as SOC cohorts, further underscoring these findings.

Prior biomarker analyses highlight the potential of TPE to rebalance pro- and anti-inflammatory responses in septic shock [39, 40] as a physiological explanation for the improved hemodynamics and microcirculation observed clinically. Importantly, TPE is distinct from other extracorporeal blood purification methods as it not only removes potentially harmful cytokines or damage-associated molecular patterns [17] but also replaces protective but consumed plasma proteins such as immunoglobulins, anticoagulant and anti-permeability factors [21, 41, 42]. TPE also has the potential to ameliorate endothelial cell and glycocalyx injury as demonstrated by rebalancing the angiopoietin disbalance and attenuating heparanase−2 deficiency in sepsis with removal of injurious endothelial glycocalyx degradation products [15, 21, 22].

Whether TPE also removes streptococcal superantigens remains unresolved. Indirect evidence for the removability of exotoxin A (SpeA) and toxic shock syndrome toxin 1 from human plasma in vitro exists, and extracorporeal removal improved survival in animal models of superantigen-mediated shock[43–45]. Whether this translates into clinically relevant clearance is debated, as the in vivo distribution of these molecules is incompletely defined and compartmentalization may render the intravascular pool accessible to apheresis only a small fraction of the total body burden [46], and superantigen concentrations have to our knowledge not been quantified before and after plasma exchange in patients.

Of note, the investigated patient collective was rather young, with few chronic comorbidities and a low rate of prior immunosuppression, which is in line with prior SRSS populations described [47]. The TPE treated group had significantly aggravated organ-dysfunction at baseline which led to the broader use of hydrocortisone as an adjunctive treatment in the TPE group, which itself may improve hemodynamics [48]. Yet, TPE was usually initiated because of deterioration in overall organ-dysfunction despite use of SOC, which included steroids.

In our study, no significant safety signals were observed with the use of TPE, similarly to previous reports [15, 16, 49]. SRSS-related complications were comparable between the groups despite significantly higher SOFA scores in the TPE-group. Although acral necroses occurred more often in the TPE group, an attribution to the vastly higher vasopressor-doses and DIC in the TPE group is likely [50].

Of note, ICU length of stay was longer in the TPE cohort, which we attribute at least in part to improved early survival, as death is a competing event that truncates length of stay in the comparator group. Sepsis-induced immunoparalysis has recently been confirmed as a targetable endotype and may predispose to secondary infection [51], but whether TPE modifies this state has not been studied and was not assessed in our cohort.

This study has several limitations. Its non-randomized, multicenter design introduces selection bias, as treatment decisions varied, TPE patients were more severely ill, had less comorbidities and TPE was available at only two of the three sites.

However, imbalances in sepsis severity, adjunctive treatment such as hydrocortisone administration and baseline comorbidities were addressed across different PSM strategies, with TPE continuously maintaining its protective effects. Attenuation of the effect estimate after accounting for hydrocortisone and clindamycin suggests that part of the association is attributable to more frequent hydrocortisone use in the TPE group.

After publication of Exchange−1, TPE became standard practice in two centers; therefore, subsequent SOC patients were excluded from PSM to minimize treatment-era related confounding. Therefore, TPE-treated patients were admitted later within the study period than their matched comparators, leaving the possibility of residual confounding by treatment era. Heterogeneity in streptococcal species, toxins, and infection foci may exist, though overall, groups were well-balanced. Finally, the relatively small number of TPE-treated patients carries a risk of model overfitting, and the cohort was not powered to assess effect modification in the subgroup of STSS patients.

While we derive from these data that TPE in SRSS is likely beneficial, the optimal selection, timing and application of TPE in septic shock patients clearly remains to be further elucidated, preferentially in ongoing (e.g. Exchange−2 (NCT05726825) [52] and PLEXIS study (NCT05093075) and future larger RCTs.

## Conclusion

In this exploratory analysis of severe streptococcal septic shock, adjunctive TPE was associated with more rapid hemodynamic stabilization and improved lactate kinetics. Adjusted survival show association with lower 90-day hospital mortality, but the retrospective design and the small, matched cohort preclude conclusions on efficacy. These hypothesis-generating findings support the prospective evaluation of TPE in SRSS, in prospective trials.

## Supplementary Information

Additional file 1.

## Data Availability

The datasets used and/or analyzed during the current study are available from the corresponding author upon reasonable request.

## Abbreviations

aHR: Adjusted hazard ratio
CI: Confidence interval
CRP: C-reactive protein
DIC: Disseminated intravascular coagulation
ECMO: Extracorporeal membrane oxygenation
HR: Hazard ratio
ICU: Intensive care unit
IQR: Interquartile range
PCT: Procalcitonin
PSM: Propensity score matching
RCT: Randomized controlled trial
SEPSIS 3: Third international consensus definitions for sepsis and septic shock
SOC: Standard of care
SOFA: Sequential organ failure assessment
spp.: Species (plural)
SRSS: Streptococcal related septic shock
STSS: Streptococcal toxic shock syndrome
TPE: Therapeutic plasma exchange
VIS: Vasoactive-inotropic score
VV-ECMO: Veno-venous extracorporeal membrane oxygenation
