# Supplementary material for: Adjunctive therapeutic plasma exchange as rescue therapy in septic shock due to streptococcal infection: a multicentric retrospective cohort study
Source: Intensive Care Med Exp. 2026 Oct 28;14:142. doi: 10.1186/s40635-026-00984-4 (PMC13719982; doi:10.1186/s40635-026-00984-4)
Supplement: Supplementary file 1 [file 40635_2026_984_MOESM1_ESM.docx]

# Supplementary Information to

**Adjunctive therapeutic plasma exchange as rescue therapy in septic shock due to streptococcal infection – a multicentric retrospective cohort study**

Jannik Ruwisch, MD^1,2,3^, Alix Buhlmann, MD^4^, Lennart Wild, MD^5^, Pedro David Wendel-Garcia, MD^4,6^, Thorben Pape, MD^1^, Daniel A Hofmaenner, MD^4^, Christian Putensen, MD^5^, Philippe Kruse^5^, Greta-Henrike Holtgrave^1^, Heiko Schenk, MD^7^, Christian Bode, MD^5^, Sascha David, MD^4^, Klaus Stahl, MD^8*^, Benjamin Seeliger, MD^1,2*^ for the Bonn-Hannover-Zurich ARDS (BonHanZA) Study Group

**Contributed equally and share last authorship*

**Authors’ affiliation(s):**

1) Department of Respiratory Medicine and Infectious Diseases, Hannover Medical School, Hannover, Germany
2) Biomedical Research in End-Stage and Obstructive Lung Disease (BREATH), Hannover Medical School (MHH), German Center for Lung Research (DZL), Hanover, Germany
3) PRACTIS Clinician Scientist Program, Dean’s Office for Academic Career Development, Hannover Medical School, Germany
4) Institute of Intensive Care Medicine, University and University Hospital Zurich, Zurich
5) Department of Anaesthesiology and Intensive Care Medicine, University Hospital Bonn, Bonn, Germany
6) Division of Cardiothoracic Anaesthesia and Intensive Care Medicine, Department of General Anaesthesia, Intensive Care and Pain Medicine, Medical University Vienna, Vienna
7) Department of Nephrology and Hypertension, Hannover Medical School, Hannover, Germany
8) Department of Gastroenterology, Hepatology, Infectious Diseases and Endocrinology, Hannover Medical School, Hannover, Germany

**Corresponding author details:**

Dr. Benjamin Seeliger, Hannover Medical School, Department of Respiratory Medicine and Infectious Diseases, Carl-Neuberg-Strasse 1, 30625 Hannover, Germany; [seeliger.benjamin@mh-hannover.de](mailto:seeliger.benjamin@mh-hannover.de)

**SUPPLEMENTARY FIGURES**

**Figure S1.** **Temporal available hemostasis indices in the full cohort.** Log-scaled platelet counts declined significantly throughout the initial 24h in the TPE and the SOC group (**A**). Difference in d-dimer levels at baseline did not reach statistical significance. Three values in the SOC group were censored at assay maximum of 20 mg/l (triangles) (**B**).

*TPE: Therapeutic plasma exchange and SOC: standard of care.*

**Figure S2.** **Sensitivity analyses alternative propensity-score specifications.** Love plots depict absolute standardized mean differences for all covariates entered the respective propensity-score model, before (grey circles) and after (black triangles) 1:1 optimal matching. The dashed line indicates the conventional balance threshold of 0.1, the dotted line the more lenient criterion of 0.25. Covariates are ordered by the extent of their imbalance before matching. Primary model as reported in the manuscript, matching for age, sex, VIS_0h_, streptococcal species group, prior immunosuppression, SOFA_0h_ and lactate_0h_. Alternative model in which the streptococcal species group was replaced by the primary site of infection and to which invasive mechanical ventilation, renal replacement therapy and VV-ECMO at baseline were added. Extended model additionally accounting for procalcitonin (log-transformed), hydrocortisone and adjunctive clindamycin therapy (**A**). SOFA_0h_ adjusted Cox regression models of 90-day hospital mortality (**B**).

*SOFA_0h_: sequential organ failure assessment at baseline; VIS_0h_: vasoactive inotropy score at baseline; lactate0h: lactate at baseline; PCT: procalcitonin; Clindam.: Clindamycin, PSM: propensity score matching; TPE: therapeutic plasma exchange; SOC: standard of care; VV-ECMO: veno-venous extracorporeal membrane oxygenation; aHR: adjusted hazard ratio; CI: confidence interval; N: number of patients.*

**Figure S3.** **Sensitivity analysis excluding the Zurich (USZ) site.** Love plots depict absolute standardized mean differences for all covariates entered the respective propensity-score model, before (grey circles) and after (black triangles) 1:1 optimal matching. The dashed line indicates the conventional balance threshold of 0.1, the dotted line the more lenient criterion of 0.25. Covariates are ordered by the extent of their imbalance before matching (**A**). Matched survival analysis showed significant improved survival in the TPE group by log-rank test (**B**). Effect size of the PSM matched cox regression models excluding the Zurich center demonstrated significant reduction in mortality in the for the TPE group (**C**).

*TPE: therapeutic plasma exchange; SOC: standard of care;* SOFA_0h_: *sequential organ failure assessment at baseline; aHR: adjusted hazard ratio; Lactate_0h_: lactate at baseline; VIS_0h_: vasoactive inotropy score at baseline; Imm. Supp/Imm.: immunosuppression at baseline; PSM: propensity score matched; N: number of patients and CI: confidence interval. #=log-rank test; ‡=SOFA_0h_ adjusted cox-regression model.*

**Figure S4.** **Survival** **sub-analysis of TPE and SOC-treated patients.**

Sankey plot illustrating the relative distribution of patients **(A)**. Final column indicates patients with decreasing VIS throughout the initial 24 hours upon admission in the TPE group. Responders are color coded in turquoise, while non-responders are color coded in brown. 90-day hospital mortality analysis demonstrated improved outcomes in the responder group, which became significant in the unadjusted site-clustered cox-regression model **(B)**. Paired VIS kinetics between 0h und 24h of TPE treated survivors (green) and non-survivors (black) depicted a significant VIS decrease in survivors but not in non-survivors **(C)**. Hazard ratio for 90-day hospital mortality was significantly increased in non-responders in site-clustered unadjusted cox-regression model **(D)**. 90-day ICU mortality analysis within SOC patients demonstrated significantly improved outcomes in the responder group (**E**), which was also significant in the unadjusted site-clustered cox-regression model (**F**). Paired VIS kinetics between 0h und 24h of SOC treated survivors (green) and non-survivors (black) depicted a significant VIS decrease in survivors but not in non-survivors (**G**). VIS levels were logarithmized. Responders are color coded in turquoise, while non-responders are color coded in brown.

*S: streptococcus; TPE: therapeutic plasma exchange; SOC: standard of care; HR: hazard ratio and VIS: vasoactive inotropy score. #=log-rank test; ‡=site clustered unadjusted cox-regression model.*

**Figure S5.** **Temporal shock indices in the propensity score matched cohort.** VIS score declined significantly throughout the initial 24h in the TPE group, but not in the SOC group (**A**). This trend was mirrored for early lactate dynamics (**B**).

*VIS: Vasoactive-inotropic score; TPE: Therapeutic plasma exchange and SOC: standard of care.*
